# Effect of Sequential Inoculation of *Tetragenococcus halophilus* and *Wickerhamomyces anomalus* on the Flavour Formation of Early-Stage Moromi Fermented at a Lower Temperature

**DOI:** 10.3390/foods12183509

**Published:** 2023-09-21

**Authors:** Xinzhi Li, Xinyu Xu, Changzheng Wu, Xing Tong, Shiyi Ou

**Affiliations:** 1 Department of Food Science and Technology, Jinan University, Guangzhou 510632, China; li.xinzhi@u.nus.edu; 2 Guangdong Haitian Innovation Technology Co., Ltd., Foshan 528000, China; 3 Key Laboratory of Advanced Technology Enterprise of Guangdong Seasoning Food Biofermentation, Foshan 528000, China; 4 Guangdong Provincial Research Centre of Brewing Microbiology Breeding and Fermentation Engineering Technology, Foshan 528000, China

**Keywords:** moromi fermentation, sequential inoculation, *Tetragenococcus halophilus*, *Wickerhamomyces anomalus*, synergism, flavour compounds

## Abstract

Microbial inoculation in moromi fermentation has a great influence on the physicochemical and flavour properties of soy sauces. This work investigated the effect of inoculating *Tetragenococcus halophilus* and *Wickerhamomyces anomalus* on the flavour formation of early-stage moromi (30 days) fermented at a lower temperature (22 °C) by determining their physicochemical and aroma changes. The results showed that single yeast or LAB inoculation increased the production of amino nitrogen, lactic acid and acetic acid, as well as free amino acids and key flavour components. Particularly, the sequential inoculation of *T. halophilus* and *W. anomalus* produced more free amino acids and aromatic compounds, and there might be synergistic effects between these two strains. More characteristic soy sauce flavour compounds, such as benzaldehyde, HEMF, guaiacol and methyl maltol were detected in the sequentially inoculated moromi, and this sample showed higher scores in savoury, roasted and caramel intensities. These results confirmed that sequential inoculation of *T. halophilus* and *W. anomalus* could be a choice for the future production of moromi with good flavour and quality under a lower temperature.

## 1. Introduction

Soy sauce is a traditional liquid condiment and seasoning ingredient in Asian countries [1]. All fermented soy sauce production shares a two-step fermentation process, namely koji (solid-state) fermentation and moromi (liquid-state) fermentation. However, due to different raw materials, starter cultures, fermentation time and temperature applied, the tastes and aromas of fermented soy sauce are varied [2,3]. Moromi fermentation (also called soy sauce mash fermentation) is the second step of soy sauce fermentation, where the koji (moulded soybeans and wheat) prepared in step one is immersed in 18–23% brine and naturally fermented at ambient temperatures (15–30 °C) and outdoor environments for 3–6 months [1,4,5]. In the early stage of moromi fermentation, the growths of halophilic yeasts and lactic acid bacteria (LAB) are facilitated, whereas the propagation of most spoilage microorganisms and pathogens is restricted [1,6]. Firstly, LAB transfers available sugars to organic acids (mainly lactic acid or acetic acid), lowering the pH of the moromi to 4.5–5.0, giving an acidified environment for the propagation of yeasts, which subsequently utilise carbohydrates and produce alcohols and other characteristic taste and aromatic compounds via various pathways [6,7].

Among the flavour-producing soy sauce microorganisms, *Tetragenococcus halophilus* and *Zygosaccharomyces rouxii* are the most prevalent and well-studied LAB and yeast, respectively [1,5]. *T. halophilus* is responsible for the production of organic acids such as lactic acid, acetic acid, formic acid and some pungent aromatic compounds including benzaldehyde and methyl acetate [1]. Also, the inoculation of *T. halophilus* in the moromi stage is reported to increase the formation of furfural, furfural alcohol, 2-hydroxy-3-methyl-2-cyclopenten-1-one and methional, and these compounds are likely to be produced in the Maillard reaction under a lower pH during moromi fermentation [1,8,9]. In addition, the metabolism of *Z. rouxii* is reported to be associated with the production of ethanol, 2,3-methylbutanol, 1-butanol, 2,5-dimethyl-4-hydroxy-3(2H)-furanone (HDMF) and 2(or 5)-ethyl-4-hydroxy-5(or 2)-methyl-3(2H)-furanone (HEMF) [9,10,11].

However, an antagonism relationship between *T. halophilus* and *Z. rouxii* is commonly observed in moromi fermentation [1]. Several studies have suggested that in traditional practice, organic acids, especially acetic acid, produced by *T. halophilus*, inhibited the growth of *Z. rouxii* by lowering the pH of the environment and further restricting the respiratory activity and cytochrome formation of *Z. rouxii*; therefore, the production of flavour compounds by the yeast was restricted [12]. As a result, the fermentation time of soy sauce was considerably long (6–12 months) to achieve the qualities required, and the fermentation efficiency was low [13,14]. Therefore, to further increase aroma formation in moromi fermentation in a shorter fermentation period, as well as develop a flavour-enhanced product, some studies reported isolating other aroma-producing yeasts, including *Wickerhamomyces* species, *Tetrapisispora blattae*, and *Torulaspora delbrueckii*, to enhance the production of alcohols and esters in moromi fermentation [4,10,15,16]. So far, most studies have reported co-inoculating *T. halophilus* and *Z. rouxii* to increase the production of characteristic soy sauce aroma; few have reported the effect of inoculating *T. halophilus* and other ester-producing yeast species on moromi fermentation [5,17,18,19].

Other than flavour-contributing microorganisms, fermentation temperature is also one of the key factors in defining the qualities of soy sauce. In regular industrial practice, the temperature of soy sauce production mainly depends on the outdoor weather; in other words, sometimes moromi fermentation is conducted under relatively low temperatures (winter time) [20,21]. Temperature downshift could affect the growth patterns and metabolic performance of microorganisms in moromi fermentation. In general, longer fermentation time is required when the fermentation is conducted in lower temperatures, which also causes decreases in production efficiency and increases production costs [22,23,24]. However, a lower fermentation temperature might be beneficial for the flavour formation of the moromi [22,23,25,26]. Wei et al. [26] reported that a lower starting temperature of 15 °C, then set at 30 °C for the remaining period of moromi fermentation, increased the sensory qualities, including the aroma profiles and the sensory scores of the fermented soy sauce. Zhou et al. [24]. found that setting an extremely low temperature of 4 °C at the initial moromi fermentation was beneficial for the flavour formation of no-salt soy sauce, resulting in more desired taste components and amino acid content produced compared to controls. Nevertheless, a longer fermentation time is needed to achieve the goal of producing high qualities of soy sauce in these studies. Therefore, the inoculation of functional microorganisms in the early stage of moromi fermentation to enhance flavour production could be a promising solution for low-temperature soy sauce fermentation.

In this study, moromi fermentation was conducted under ambient temperatures (recorded average temperature was about 20–22 °C) in the laboratory, to simulate the actual soy sauce production during the winter period. *T. halophilus* and *W. anomalus* were selected as the flavour-enhancing strains and inoculated in the early stage (the first 0–5 days) of moromi fermentation to enhance their influences on flavour production. *T. halophilus* was firstly inoculated at the beginning to start lactic acid fermentation and lower the pH of the environment, then *W. anomalus* was sequentially inoculated on the 5th day to enhance alcoholic fermentation and ester production. The fermentation was terminated on the 30th day. The effect of culture inoculation on the flavour formation in moromi fermentation was evaluated by determining the changes in physicochemical properties and representative key volatile compounds of the fermented moromi samples. The knowledge obtained in this study could bring new ideas to the food industry for producing high-quality soy sauce with enhanced flavour in wintertime.

## 2. Materials and Methods

### 2.1. Culture Preparation

Lactic acid bacteria (LAB) strain *Tetragenococcus halophilus* and yeast strain *Wickerhamomyces anomalus* were isolated and identified from the high-salt liquid-state soy sauce moromi samples previously and stored at the culture centre of Guangdong Haitian Innovation Tech Co., Ltd. (Foshan, China). Before fermentation, the yeast strain was activated in yeast-malt broth (Oxoid, Basingstoke, UK) supplemented with 5% (*w*/*w*) of sodium chloride at 30 °C for 48–72 h, and the LAB strain was transferred to MRS (De Man, Rogosa and Sharpe) broth (Oxoid, Basingstoke, UK) with 5% (*w*/*w*) of sodium chloride addition and incubated at 37 °C for 48 h.

### 2.2. Moromi Fermentation

The koji used for moromi fermentation was prepared using soybean and wheat flour with *Aspergillus oryzae* 3.042 spores, which was provided by Foshan Haday Flavoring & Food Co., Ltd. (Foshan, China). The matured koji was transferred to the laboratory at 40–45 h and immersed in saturated brine solution (m/m = 1:2) to make a final sodium chloride concentration of 15% (*w*/*w*). The mixed moromi samples were placed in a 2-liter tank with a loosened cap and incubated for a 30-day period at ambient temperatures around 20–22 °C.

### 2.3. Microbial Inoculation

The activated *T. halophilus* and *W. anomalus* cells were first washed with 0.85% (*w*/*w*) saline solution and centrifuged at 8000× *g* for 10 min twice, and the cell pellets were re-suspended with saline water to obtain approximately cell counts of 10^6^–10^7^ CFU/g. There were four types of inoculation strategies: (i) control: non-inoculated moromi; (ii) T0: with single *T. halophilus* inoculated at day 0 of moromi fermentation (inoculated cell counts were about 10^5^ CFU/g moromi sample); (iii) W5: with *W. anomalus* inoculated at day 5 of moromi fermentation (inoculated cell counts were about 10^5^ CFU/g sample); and (iv) T0W5: with single *T. halophilus* inoculated at day 0 (10^5^ CFU/g) and *W. anomalus* inoculated at day 5 of moromi fermentation (10^5^ CFU/g). Sampling was conducted on day 0, 5, 15, and 30, in which approximately 15–20 g of moromi samples were taken out from the tanks, and all taken samples were first filtered through filter papers before analysis.

### 2.4. Physicochemical Analysis

The pH value of each filtered moromi sample was measured using a pH meter (Mettler-Toledo GmbH, Greifensee, Switzerland). The contents of total titratable acids (TA), amino nitrogen (AN), total nitrogen (TN), and reducing sugar (RS) were determined, referring to the methods described in Liu et al. [5]. In brief, TA content was measured using an automatic potentiometric titrator (model 905, Metrohm, Herisau, Switzerland), and the amount of NaOH (0.05 M, Sigma-Aldrich, St. Louis, MO, USA) used in the titration was recorded for TA calculation; AN content was determined using formaldehyde titration by measuring the NaOH (0.1 M, Sigma-Aldrich, St. Louis, MO, USA) consumed, and the titration was also performed by the same titrator; TN content was measured using the method according to the method described in Cui et al. [22]; RS content was determined by the modified DNS method described in Li et al. [27]. 

### 2.5. Organic Acids and Free Amino Acids Analysis

The organic acid content of the filtered moromi samples was analysed using high-performance liquid chromatography (HPLC, Agilent 1290 Infinity, CA, USA) according to the methods and setting in Li et al. [28]. The samples were first filtered through a 0.2 μm Minisart RC 15 syringe filter (Sartorius, Goettingen, Germany) and mixed with 60% (*w*/*w*) trichloroacetic acid (Sigma-Aldrich, St. Louis, MO, USA) in the ratio of 1:1 (*w*/*w*) to allow protein precipitation for 1–2 h. Then, the samples were centrifuged at 10,000× *g* at 4 °C for 15 min to remove the precipitate, and the supernatant collected was ready for injection. In this study, lactic acid, citric acid, acetic acid and malic acid were identified and quantified with a series of external standards (Sigma-Aldrich, St. Louis, MO, USA).

In addition, the collected supernatant was also used for free amino acids analysis according to Gao et al. [29] using an amino acid analyser (LA8080, Hitachi, Tokyo, Japan). Briefly, a lithium-cation exchange column was used along with ninhydrin (a reagent for post-column derivatisation) and eluents (Eluent A to Eluent F) provided by the manufacturer. The sixteen targeted free amino acids were detected at the wavelength of 570 nm, and proline (Pro) was detected at 440 nm. The identification and quantification of these amino acids was performed using the calibration factors that were generated by measuring the concentrations of the amino acid standards (Thermo Fisher Scientific, Waltham, MA, USA) according to the manufacturer’s instructions.

### 2.6. Key Volatile Compound Analysis

The volatile compounds of both raw moromi samples (non-fermented control) and 30-day fermented moromi samples were extracted and analysed according to the method described in Gao et al. [29] with some modifications. Briefly, 10.0 μL of internal standard 2-octanol (Sigma-Aldrich, St. Louis, MO, USA) was mixed with 5.0 g of filtered samples to obtain a final concentration of 0.04 mg/mL of 2-octanol. The mixtures were kept in a 20 mL glass vial sealed with PTFE septum (Sartorius, Goettingen, Germany) before extraction. The volatile was extracted by an 85 μm carboxy/polydimethylsiloxane SPME fibre (CAR/PDMS, Supelco, Bellefonte, PA, USA) for 30 min at 60 °C under 250 rpm agitation using a Combi Pal autosampler (CTC Analytics, Zwingen, Switzerland). Compound separation and analysis were performed using a GC-MS (model 7890B-5977B, Agilent, Santa Clara, CA, USA) with an HP-INOWAX capillary column (60 m length, 0.25 mm i.d., 0.25 µm film thickness, Agilent). Helium was used as the carrier gas with a flow rate of 1.2 mL per minute. The oven temperature was first held at 40 °C for 3 min, then increased to 100 °C at a rate of 5 °C per min, then increased to 220 °C at a rate of 60 °C per min with a holding time of 10 min.

Volatiles were identified by matching the NIST 8.0 and Wiley 275 databases with their mass spectra (MS) and verified by comparing their linear retention index (LRI) with literature data in the NIST WebBook. The semi-quantification of each compound was performed using the GC-MS peak areas and the internal standard (2-octanol), and the volatile compounds were expressed in μg/L. Data were reported in mean values with standard deviations (*n* = 3).

### 2.7. Sensory Evaluation

The sensory evaluation was conducted by referring to the method described in Liu et al. [5]. A total of 23 panellists (12 men and 11 women aged from 25 to 40 years old) from Guangdong Haitian Innovation Tech Co., Ltd. (Foshan, China) were invited to the sensory section; these were professional flavourists who had been trained in soy sauce sensory evaluation in the company for at least 2 years due to their requirement of work. Sensory evaluation was performed in the sensory panel room at 25 ± 2 °C for 3 different sessions. A series of five aqueous solutions of soybean paste (100,000 μg/L), ethanol (700,000 μg/L), 4-ethyl-2-methoxyphenol (231 μg/L), HEMF (140 μg/L) and ethyl acetate (85,848 μg/L), which represented “savoury, alcoholic, roasted, caramel and floral”, respectively, were served as the standard of aroma the with gradient intensities according to the study of Liu et al. [5]. The panellists were required to score the aroma intensities on a line scale from 0 (lowest intensity) to 5 (highest intensity).

### 2.8. Statistical Analysis

This study was conducted using a randomized complete block design with three independent blocks and four treatments, as indicated. All data reported in this study were obtained from three independent experiments with duplicate samplings; in addition, this study was conducted as a randomized complete block design with 3 independent blocks and 4 treatments, as indicated (*n* = 6). All data are expressed in mean values and standard deviations (SD). One-way ANOVA combined with Duncan’s test were performed to compare the mean values. Differences were considered statistically significant when *p* ≤ 0.05.

## 3. Results and Discussion

### 3.1. Physicochemical Changes

**Figure 1:**
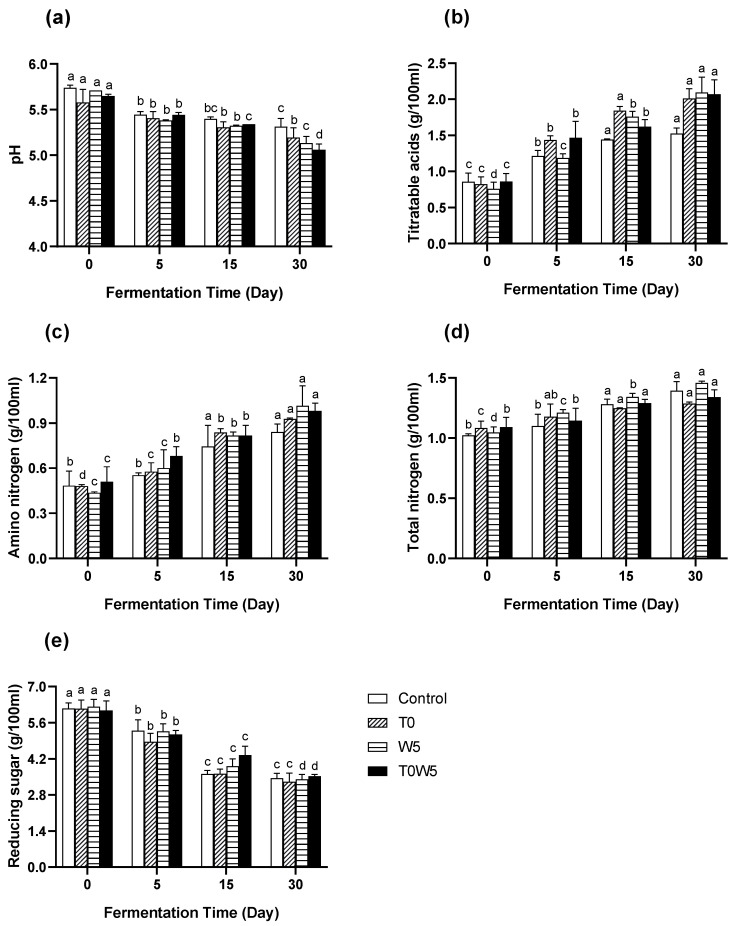
Changes in (**a**) pH, (**b**) titratable acidity, (**c**) amino nitrogen, and (**d**) total nitrogen and (**e**) reducing sugar during moromi fermentation. ^a–d^ Values within the same sample on different days followed by the same letters are not significantly different (*p* > 0.05). Data were expressed in g/100 mL (*n* = 3).

As depicted in Figure 1a, under a lower fermentation temperature, the pH of the non-inoculated control and inoculated samples only gradually decreased from around 5.5–5.7 to 5.1–5.3 on the 30th day. The inoculation of *T. halophilus* and *W. anomalus* slightly accelerated the decrease rate of the pH in moromi fermentation, with sample T0W5 presenting the lowest pH of 5.06 on the 30th day. The accelerated decrease in pH in the culture-inoculated samples could be due to the enhanced production of organic acids (mainly lactic acid and acetic acid) by the inoculated strains [5]. Also, the accumulation of fatty acids and amino acids could acidify and lower the pH of the moromi [30]. The changes in the titratable acidity (TA) were in accordance with the changes in the pH values. As shown in Figure 1b, culture-inoculated samples increased the production and accumulation of organic acids in moromi fermentation. The TA content of the moromi samples was about 0.8 g/100 mL before fermentation, and rapidly increased to approximately 2.0 g/100 mL for culture-inoculated samples at the end of fermentation, where the ending point of TA content in the non-inoculated control was only about 1.5 g/100 mL.

Amino nitrogen (AN) is one of the key factors that define the quality of fermented soy sauce, and it is also often used in determining the endpoint of soy sauce fermentation [31]. As shown in Figure 1c, the AN content increased from the original 0.5 g/100 mL to about 0.7–0.9 g/100 mL at the end of fermentation. The highest AN content was found in W5 of 1.01 g/100 mL, followed by T0W5 and T0, accounting for 0.98 and 0.93 g/100 mL, respectively. The AN content continuously increased during fermentation because of protein degradation by microbial activities [32]. In this stage, the Maillard reaction that consumes amino acids and amino nitrogen was relatively inactive due to the low temperature, resulting in the accumulation of amino nitrogen [33]. In China, fermented soy sauce with a free amino-type nitrogen content of no less than 0.8 g/100 mL is considered premium-grade soy sauce [5,31]. The culture inoculation in the present study increased the amino nitrogen production as the newcomers rapidly utilised the nutrients in the environments and the rapid degradation of proteins of raw materials resulted in the quick production of amino nitrogen [34,35]. At this rate, the fermented moromi showed great potential of becoming a premium-grade soy sauce when a longer fermentation time was applied. However, different inoculation strategies showed little difference in increasing the amino nitrogen content, which agreed with the results reported by Zhang et al. [36], where the authors found that the co-addition of LAB and yeast in soy sauce fermentation showed no significant effect on the productions of amino nitrogen.

Figure 1d demonstrated the changes in total nitrogen (TN) content in the fermented moromi samples, rising from around 1.0 g/100 mL at day 0 to about 1.4 g/100 mL at day 30 for all samples. A higher TN content was found in control and W5, accounting for 1.40 and 1.45 g/100 mL, respectively; the lowest TN content was found in T0 at 1.29 g/100 mL. The different increases in TN content indicated that the production and consumption of soluble nitrogen might be dependent on the strains inoculated. The addition of *T. halophilus* could enhance the absorption and transformation of soluble nitrogen in the moromi, resulting in a lower TN content being detected. However, these results were contrary to the studies of Liu et al. [5] and Zhang et al. [36], where the researchers found that the co-addition of *T. halophilus* and *Z. rouxii* showed little difference in changing the TN content of fermented moromi. Since *W. anomalus* was mainly used for producing ethyl acetate in wine and liquor fermentation, the effects of co-adding *W. anomalus* and *T. halophilus* on moromi fermentation may be different from those adding *Z. rouxii* instead, but the actual mechanism behind this would need to be further explored [37,38].

The changes from reducing the sugar (RS) content in moromi fermentation are presented in Figure 1e. By the 30th day, the RS content was reduced significantly (*p* < 0.05) to approximately 3.5 g/100 mL from 6.0 g/100 mL (day 0). *W. anomalus*-inoculated samples showed less sugar consumption, with T0W5 showing the slowest sugar consumption rate during the fermentation, followed by W5. On the one hand, the slow consumption rate of sugars in the present study was likely due to the lower fermentation temperature, because, in an appropriate fermentation temperature (28–32 °C), sugars were mostly utilised within 30 days in moromi fermentation [30]. On the other hand, *W. anomalus* fermentation utilised fewer sugars and produced a large amount of esters and alcohols, which had a strong fruit flavour [38].

### 3.2. Changes in Organic Acids

**Figure 2:**
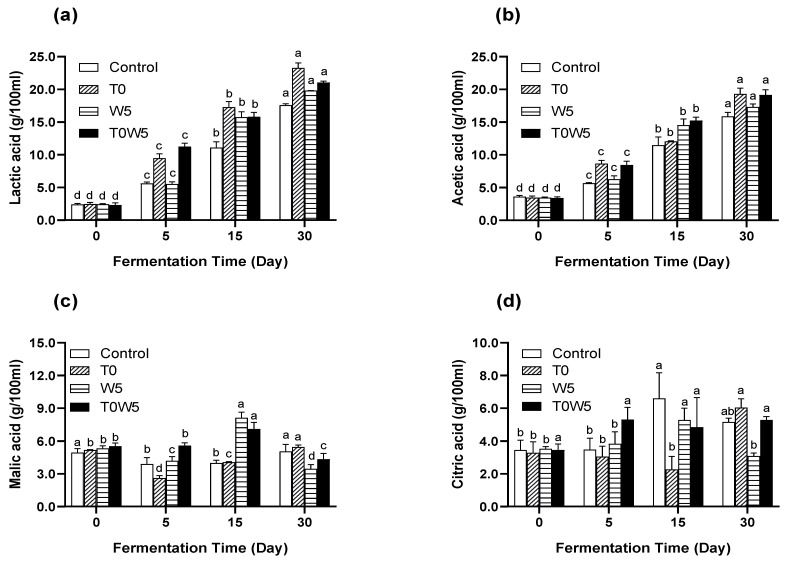
Changes in (**a**) lactic acid, (**b**) acetic acid, (**c**) malic acid, and (**d**) citric acid concentrations in moromi fermentation. ^a–d^ Values within the same sample on different days followed by the same letters are not significantly different (*p* > 0.05). Data were expressed in g/100 mL (*n* = 3).

Organic acid production in moromi fermentation could affect the flavours of the soy sauce, and they are also important metabolites that indicate the microbial activities in the fermentation [5]. As shown in Figure 2a,b, lactic acid and acetic acid gradually increased and accumulated in the fermentation. The maximum lactic acid production was found in T0, accounting for 23.3 g/100 mL at day 30. T0W5 and W5 had lactic acid production of 21.1 and 19.8 g/100 mL, respectively. In addition, the highest acetic acid content was found in sample T0, accounting for 19.3 g/100 mL at day 30, followed by T0W5, accounting for 19.1 g/100 mL. Previous studies showed that the production of lactic acid could improve the taste of fermented soy sauce by neutralising the saltiness of soy sauce, making the flavour more refreshing, whereas the formation of acetic acid, as a volatile fatty acid, contributes to the sour smell of soy sauce [5,39,40,41]. The inoculation of *W. anomalus* accelerated the production of lactic acid, which could be beneficial to flavour formation in moromi fermentation.

Significantly, the malic acid content in W5 and T0W5 was almost two times higher than control and T0 on the 15th day (Figure 2c), accounting for 7.0–8.0 g/100 mL; simultaneously, the citric acid content of the control was almost three times higher than sample T0 at the 15th day (Figure 2d), accounting for 6.2 g/100 mL. The consistent malic acid production might reflect the continually fermenting process of yeasts, as malic acid is one of the key intermediary products of the tricarboxylic acid (TCA) cycle, and this result suggested that *W. anomalus* was very active in sugar metabolism [29,38]. On the other hand, Li et al. [28] found that LAB could produce succinic acid from citric acid via the reductive pathway of the TCA cycle, and acetic acid may be formed from citrate metabolism by citrate lyase. Therefore, the contents of malic acid and citric acid were fluctuant throughout moromi fermentation, and their changing patterns varied among different inoculated samples mainly due to different microbial metabolism patterns. Since malic acid and citric acid are mainly formed via the tricarboxylic acid (TCA) cycle of microbial metabolism, these results indicated that the inoculation of *T. halophilus* and *W. anomalus* enhanced the metabolism of microorganisms by increasing the production of organic acids during moromi fermentation. However, different inoculation strategies might lead to different organic acid production patterns, because of the complex natural environment and complicated interactions among endogenous and exogenous strains [2,5,9,10].

### 3.3. Changes in Free Amino Acids

As shown in Table 1, a total of 17 free amino acids were determined in the non-inoculated control (day 0 and day 30) and inoculated moromi samples. The content of amino acids in all samples significantly increased after fermentation, and T0W5 produced the maximum amount of total amino acids, accounting for 79.75 μg/100 mL. The single or co-inoculation of *T. halophilus* and *W. anomalus* enhanced the hydrolysis of protein by producing more amino acids in moromi fermentation. These results were in line with the studies of Liu et al. [5] and Sun et al. [42], as *T. halophilus* and *W. anomalus* were commonly used as starter cultures in soy sauce and wine fermentation, respectively, and both cultures demonstrated promising acid production and proteolytic activity. The total amount of amino acid contents in T0 and W5 showed no significant differences, indicating that the proteolytic activities of these two stains might be equivalent. However, unlike the results in Liu et al. [5], an antagonism relationship was found in *T. halophilus* and *Z. rouxii* and the co-inoculation of *T. halophilus* and *W. anomalus* increased the production of amino acids, suggesting that there might be a synergism relationship between these two strains. As *T. halophilus* produced organic acids and lowered the pH of the moromi in the early fermentation, a lower pH might be beneficial to the growth of *W. anomalus* instead [38,42].

Umami amino acids—aspartic acid (Asp) and glutamic acid (Glu)—increased the most in T0W5, accounting for 8.22 μg/100 mL after fermentation, which would be favourable to the taste of the fermented soy sauce [5,17,18]. In addition, T0W5 produced the highest amount of threonine (Thr, 6.22 μg/100 mL), serine (Ser, 4.92 μg/100 mL), glycine (Gly, 3.17 μg/100 mL), alanine (Ala, 4.42 μg/100 mL), and proline (Pro, 3.26 μg/100 mL), which are contributing “sweet” tastes in the food products [28,31]. The favourable increases in “umami” and “sweet” amino acids in T0W5, indicating the production of amino acids might be stimulated when *T. halophilus* and *W. anomalus* were mixed-inoculated in the moromi.

### 3.4. Changes in Volatile Compounds

The key volatile compounds of non-fermented controls and fermented samples were classified into alcohols, acids, aldehydes, esters, and phenols, as presented in Table 2. Overall, the detected volatile compounds showed an increase after fermentation to different extents, though the types and amount of volatile compounds were not as much as those moromi fermented under higher temperatures (e.g., 30 °C and 40 °C) [3,5,7,30,38]. The culture inoculation increased the production of the volatile compounds compared to non-inoculated controls [5,13,17]. Ethanol was the most dominant compound detected in all fermented samples, especially in W5-30 and T0W5-30, accounting for 591.8 and 568.5 μg/L, respectively. Ethanol is mainly formed from the anaerobic fermentation by yeasts, and contributes to the characteristic odour of “alcoholic” in fermented soy sauce [2,17]. Previous studies found that most yeasts, such as *Z. rouxii* or *W. anomalus* produce more ethanol than LAB (i.e., *T. halophilus*) in moromi fermentation [5,17,36]. In the present study, the addition of *W. anomalus* increased ethanol production, resulting in a significantly higher amount of ethanol detected in W5-30 and T0W5-30. In addition, the production of 2-phenylethanol was also increased in W5-30 and T0W5-30. 2-Phenylethanol is commonly found in traditional Chinese soy sauce as a typical fragrant compound, giving the product a sweet and honey-like odour [34]. The formation of 2-phenylethanol is associated with the Ehrlich pathway of yeast metabolism [43]. In addition, the volatile acetic acid was significantly increased after fermentation due to the enhanced metabolism of inoculated LAB and yeasts, though the volatile acids content in the fermented moromi samples accounted for only 2.7–4.1% of all the volatile compounds detected. The acetic acid formation might be related to the enzymatic oxidation of acetaldehyde from pyruvate decarboxylation in yeast or LAB cells [6,9]. The low volatile acid production in this study might be due to the restriction of enzymatic activities under a lower fermentation temperature [23,30].

Benzaldehyde is considered one of the crucial aromatic volatile aldehydes that give soy sauce a characteristic burnt and caramel aroma [5,36]. In this study, culture inoculation might have stimulated the formation of benzaldehyde in moromi fermentation, with T0W5 having the highest amount of benzaldehyde detected (24.19 μg/L). The formation of benzaldehyde mainly originated from the degradation of amino acids (mainly phenylalanine) through the Ehrlich pathway in yeast metabolism [13,14,27,28]. In addition, 3-methyl-thiopropionaldehydethe (cooked potato) was detected as the highest in T0W5-30 (9.64 μg/L), indicating the potential high production of methionol (a typical soy and garlic aroma) in the following moromi fermentation if longer fermentation time is applied [25,36].

The esterification of alcohols and acids in yeast metabolism is the most common pathway for ester formation [5,27,28]. Esters are considered one of the most distinct aromatic compounds in yeast-fermented foods, as human noses are very sensitive to volatile esters [5]. In the present study, ethyl isobutyrate (sweet), methyl phenylacetate (honey) and ethyl palmitate (milky) were detected largely increased in *W. anomalus*-inoculated samples (W5 and T0W5), giving a sweet and honey-like note to the fermented moromi. *W. anomalus* is known to synthesize high-activity enzymes and transform the precursor materials into esters, acids, and higher alcohols while causing weak alcohol resistance [38,42]. In the present study, sequentially inoculating *T. halophilus* and *W. anomalus* enhanced the fermentation performance of *W. anomalus* in moromi fermentation.

The amount of 4-hydroxy-2 (or 5)-ethyl-5 (or 2)-methyl-3 (2H)-furanone (HEMF, roasted and burnt) was below detection limits for non-inoculated controls but found in T0-30, W5-30, and T0W5-30, accounting for 5.74, 3.75, and 9.93 μg/L, respectively. Previous studies reported that the production of HEMF was related to the metabolism of *Z. rouxii*, and delaying the inoculation of *Z. rouxii* to moromi samples could significantly increase the formation of HEMF [5,12,17]. These studies indicated a potential competition between *Z. rouxii* and *T. halophilus*, and suggested that their concurrent inoculation might hinder the production of HEME [2]. However, in this study, both the single and sequential inoculation of *T. halophilus* and *W. anomalus* showed increased production of HEMF, again suggesting a synergistic effect between these two strains as mentioned in Section 3.3, but a deeper level of microbial interaction mechanism is unknown as of yet.

### 3.5. Sensory Evaluation

**Figure 3:**
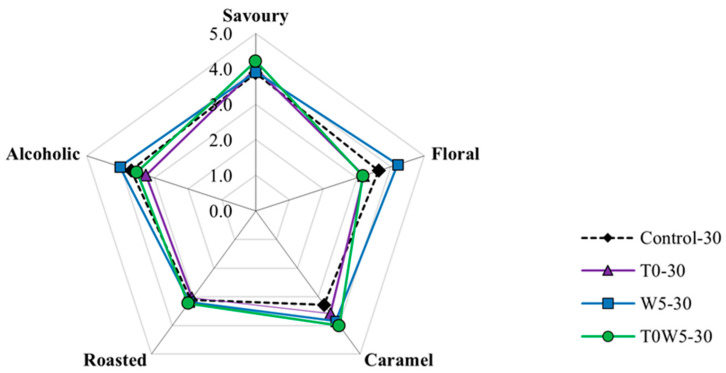
Sensory scores of different aroma intensities of the 30-day fermented moromi filtered samples.

The aroma characters of the fermented moromi samples were evaluated and scored as described in Section 2.7. As shown in Figure 3, the sequential inoculation of *T. halophilus* and *W. anomalus* (T0W5-30) enhanced the caramel, savoury, and roasted intensity scores by 22.3%, 8.8%, and 3.9%, respectively, compared to the non-inoculated control. However, T0W5 recorded lower floral and alcoholic intensity scores compared to the control. T0-30 has the lowest scores in floral and alcoholic intensities, whereas W5-30 increased both roasted and alcoholic intensity scores by 15.3% and 17.4% compared to the control. As discussed in Section 3.4, the increased production of phenols, such as HEMF, guaiacol, and methyl maltol in T0W5-30 might contribute to the increased caramel intensity, and the production of esters and alcohols increased the floral and alcoholic aroma in W5-0 [7,14]. Previous studies have related the productions of HEMF and *Z. rouxii* metabolisms during the Maillard reaction in moromi fermentation, suggesting that the inoculation of *Z. rouxii* might have enhanced the formation of HEMF [17,18,19]. In this study, we found that the synergistic relationship between inoculated *W. anomalus* and *T. halophilus* might also stimulate the metabolism activities of the original yeasts (such as *Z. rouxii*) to form more flavour components. This phenomenon was also reported by Chen et al. [38], in which the authors suggested that *W. anomalus* could provide nutrients for co-inoculated *Saccharomyces cerevisiae* in the later stage of fermentation, causing a higher production of flavour components in rice wine fermentation.

## 4. Conclusions

The sequential inoculation of *T. halophilus* and *W. anomalus* in the early stage of moromi under a lower fermentation temperature (22 °C) enhanced the flavour formation by producing more free amino acids and characteristic aromatic compounds. A synergistic relationship was observed between eaters-producing yeast *W. anomalus* and halophilic LAB *T. halophilus*, as *W. anomalus* may have grown better in a lower pH environment, which was created by acid-producing *T. halophilus*. Furthermore, the metabolites of *W. anomalus* might also provide nutrients for the growths of original moromi yeasts such as *Z. rouxii*, resulting in a more characteristic soy sauce flavour compound formation (i.e., benzaldehyde, HEMF, guaiacol, and methyl maltol). The sequentially inoculated *T. halophilus-* and *W. anomalus*-fermented moromi samples had higher sensory scores in savoury, roasted, and caramel intensities. Overall, the results of this study suggested that sequentially inoculating *T. halophilus* and *W.anomalus* could be a potential application for flavour-enhanced soy sauce production under lower fermentation temperatures in the food industry. However, this study was conducted only in laboratory-scale fermentation tanks. More practical trials should thus be carried out in future studies before this application could be used in industrial-scale soy sauce production.

## Figures and Tables

**Table 1:** Changes in free amino acid content in unfermented control and fermented moromi samples (μg/100 mL).

Groups	Amino Acids	Unfermented Control	Fermented Samples (after 30 Days of Fermentation)			
		Control-0	Control-30	T0-30	W5-30	T0W5-30
Umami	Asp	1.83 ± 0.11 c	7.04 ± 0.04 b	7.75 ± 0.38 a	7.88 ± 0.98 a	8.22 ± 1.03 a
Umami	Glu	4.15 ± 0.02 c	11.82 ± 0.76 b	13.65 ± 1.98 a	14.56 ± 1.90 a	14.46 ± 1.19 a
	Subtotal	5.98	18.86	21.40	22.44	22.68
	Percentage (%)	20.94	30.29	28.92	30.77	28.44
Sweet	Thr	2.08 ± 0.06 d	4.69 ± 0.27 c	5.78 ± 0.83 b	4.89 ± 0.54 c	6.22 ± 0.75 a
Sweet	Ser	1.74 ± 0.22 c	4.41 ± 0.22 b	4.61 ± 0.76 b	4.37 ± 0.82 b	4.92 ± 0.67 a
Sweet	Gly	0.77 ± 0.12 d	2.64 ± 0.12 c	2.98 ± 0.32 ab	2.96 ± 0.42 b	3.17 ± 0.84 a
Sweet	Ala	1.65 ± 0.42 d	3.62 ± 0.17 c	4.15 ± 0.49 ab	3.93 ± 0.78 b	4.42 ± 0.66 a
Sweet	Pro	1.93 ± 0.15 d	2.91 ± 0.23 b	2.15 ± 0.02 c	3.38 ± 0.01 a	3.26 ± 0.13 a
	Subtotal	8.17	18.27	19.67	19.53	21.99
	Percentage (%)	28.61	29.34	26.58	26.78	27.57
Bitter	Cys	0.38 ± 0.05 d	0.76 ± 0.03 c	0.88 ± 0.16 b	0.84 ± 0.09 b	0.95 ± 0.31 a
Bitter	Val	1.68 ± 0.43 d	4.07 ± 0.19 c	4.68 ± 0.51 ab	4.44 ± 0.42 b	4.97 ± 0.28 a
Bitter	Met	0.58 ± 0.03 d	1.17 ± 0.09 c	1.28 ± 0.05 b	1.27 ± 0.59 b	1.37 ± 0.09 a
Bitter	Ile	1.42 ± 0.01 d	4.09 ± 0.20 c	4.32 ± 0.08 bc	4.47 ± 0.69 ab	4.62 ± 0.16 a
Bitter	Leu	2.41 ± 0.01 d	5.10 ± 0.03 c	6.54 ± 0.91 b	6.35 ± 0.87 b	6.96 ± 0.29 a
Bitter	Tyr	0.83 ± 0.03 b	0.64 ± 0.07 c	1.01 ± 0.19 a	0.98 ± 0.04 a	1.02 ± 0.21 a
Bitter	Phe	2.09 ± 0.29 d	2.73 ± 0.11 c	4.19 ± 0.37 ab	3.99 ± 0.52 b	4.43 ± 0.45 a
Bitter	Lys	2.32 ± 0.51 e	3.59 ± 0.02 d	5.32 ± 0.83 b	4.93 ± 0.31 c	5.66 ± 0.37 a
Bitter	His	0.82 ± 0.04 d	1.77 ± 0.05 c	2.01 ± 0.09 b	1.95 ± 0.04 b	2.22 ± 0.23 a
Bitter	Arg	1.88 ± 0.20 c	1.22 ± 0.06 d	2.69 ± 0.15 b	1.74 ± 0.01 c	2.88 ± 0.24 a
	Subtotal	14.41	25.14	32.92	30.96	35.08
	Percentage (%)	50.46	40.37	44.49	42.45	43.99
	Total	28.56 ± 1.24	62.27 ± 3.32	73.99 ± 2.08	72.93 ± 3.14	79.75 ± 1.98

Different letters in the same row indicate significant differences at *p* ≤ 0.05. Values are the mean ± standard deviation of three independent replicates (*n* = 3).

**Table 2:** Changes in the relative concentration of the key volatile compounds in unfermented control and fermented moromi samples (μg/L).

LRI *	Volatile Compounds	Identification Method	Unfermented Control	Fermented Samples			
			Control-0	Control-30	T0-30	W5-30	T0W5-30
Alcohols							
893	Methanol	MS, LRI	ND	59.67 ± 6.84 a	52.99 ± 7.85 b	49.77 ± 3.05 bc	47.47 ± 2.64 c
912	Ethanol	MS, LRI	159.17 ± 5.41 c	491.69 ± 13.51 b	509.91 ± 18.72 b	591.81 ± 4.36 a	568.45 ± 11.25 a
1439	1-Octen-3-ol	MS, LRI	ND	29.43 ± 0.84 c	32.82 ± 0.13 b	36.68 ± 12.34 a	38.86 ± 0.67 a
1911	2-Phenylethanol	MS, LRI	0.63 ± 0.08 d	11.23 ± 3.66 c	17.17 ± 0.16 b	18.29 ± 0.03 b	20.63 ± 1.36 a
	Subtotal		159.80	592.02	612.89	696.55	675.41
	Percentage (%)		95.51	89.87	85.27	84.42	78.72
Acids							
1445	Acetic acid	MS, LRI	ND	18.38 ± 3.31 d	23.37 ± 0.43 c	25.37 ± 2.10 b	35.47 ± 2.78 a
	Subtotal		ND	18.38	23.37	25.37	35.47
	Percentage (%)		ND	2.79	3.25	3.07	4.13
Aldehydes							
915	2-Methylbutyraldehyde	MS, LRI	6.02 ± 1.73 d	9.28 ± 0.42 c	18.79 ± 1.25 b	18.62 ± 0.72 b	20.37 ± 0.57 a
922	Isovaleraldehyde	MS, LRI	1.50 ± 2.54 d	9.48 ± 0.97 c	18.90 ± 1.08 b	19.43 ± 1.07 b	25.29 ± 1.31 a
1177	3-Methylthiopropionaldehyde	MS, LRI	ND	4.29 ± 0.41 d	5.31 ± 0.31 c	6.46 ± 1.13 b	9.64 ± 0.21 a
1515	Benzaldehyde	MS, LRI	ND	10.45 ± 2.20 d	15.23 ± 1.41 c	18.08 ± 2.11 b	24.19 ± 1.06 a
	Subtotal		7.52	33.5	58.23	62.59	79.49
	Percentage (%)		4.49	5.09	8.10	7.59	9.27
Esters							
960	Ethyl isobutyrate	MS, LRI	ND	0.59 ± 0.18 d	0.93 ± 0.09 c	1.15 ± 0.12 b	2.22 ± 0.23 a
1755	Methyl phenylacetate	MS, LRI	ND	1.89 ± 0.22 c	1.98 ± 0.17 c	4.89 ± 0.07 b	6.79 ± 0.17 a
2224	Ethyl palmitate	MS, LRI	ND	5.93 ± 5.16 c	6.09 ± 1.72 c	15.24 ± 0.55 b	21.13 ± 1.42 a
	Subtotal		ND	8.41	9.00	21.28	30.14
	Percentage (%)		ND	1.28	1.25	2.58	3.51
Phenols							
1940	HEMF **	MS, LRI	ND	ND	5.74 ± 0.08 b	3.75 ± 0.05 c	9.93 ± 0.68 a
1862	Guaiacol	MS, LRI	ND	2.47 ± 0.27 d	5.48 ± 0.25 c	7.35 ± 0.26 b	12.06 ± 0.05 a
1988	Methyl maltol	MS, LRI	ND	3.96 ± 2.64 c	4.02 ± 0.94 c	8.21 ± 0.53 b	15.45 ± 0.82 a
	Subtotal		ND	6.43	15.24	19.31	37.44
	Percentage (%)		ND	0.98	2.12	2.34	4.36
Total			167.32	658.74	718.73	825.1	857.95

* LRI: linear retention index; ** 4-Hydroxy-2 (or 5)-ethyl-5 (or 2)-methyl-3 (2H)-furanone; Different letters in the same row indicate significant differences at *p* ≤ 0.05. Values are the mean ± standard deviation of three independent replicates (*n* = 3).

## Data Availability

The data that support the findings of this study are available from the corresponding author upon reasonable request.
